# CRISPR-Cas-Based Adaptive Immunity Mediates Phage Resistance in Periodontal Red Complex Pathogens

**DOI:** 10.3390/microorganisms11082060

**Published:** 2023-08-11

**Authors:** Pradeep Kumar Yadalam, Deepavalli Arumuganainar, Raghavendra Vamsi Anegundi, Deepti Shrivastava, Sultan Abdulkareem Ali Alftaikhah, Haifa Ali Almutairi, Muhanad Ali Alobaida, Abdullah Ahmed Alkaberi, Kumar Chandan Srivastava

**Affiliations:** 1 Department of Periodontics, Saveetha Institute of Medical and Technical Sciences (SIMATS), Saveetha Dental College and Hospitals, Saveetha University, Chennai 600077, India; arvamsi2009@gmail.com; 2 Department of Periodontics, Ragas Dental College and Hospital, 2/102, East Coast Road, Uthandi, Chennai 600119, India; deeps.271@gmail.com; 3 Periodontics Division, Preventive Dentistry Department, College of Dentistry, Jouf University, Sakaka 72345, Saudi Arabia; 4 College of Dentistry, Jouf University, Sakaka 72345, Saudi Arabia; sultan.abdulkarim.alfatakh@jodent.org (S.A.A.A.); dr.haifa.ali@jodent.org (H.A.A.); 5 General Dentist, Ministry of Health, Riyadh 12613, Saudi Arabia; maalobaida@moh.gov.sa (M.A.A.); aalkaberi@moh.gov.sa (A.A.A.); 6 Oral Medicine & Maxillofacial Radiology Division, Department of Oral & Maxillofacial Surgery & Diagnostic Sciences, College of Dentistry, Jouf University, Sakaka 72345, Saudi Arabia; drkcs.omr@gmail.com; 7 Department of Oral Medicine and Radiology, Saveetha Dental College, Saveetha Institute of Medical and Technical Sciences, Saveetha University, Chennai 602105, India

**Keywords:** CRISPR, periodontal disease, red complex bacteria, periodonitis, dysbiosis

## Abstract

Periodontal diseases are polymicrobial immune–inflammatory diseases that can severely destroy tooth-supporting structures. The critical bacteria responsible for this destruction include red complex bacteria such as *Porphoromonas gingivalis*, *Tanerella forsythia* and *Treponema denticola*. These organisms have developed adaptive immune mechanisms against bacteriophages/viruses, plasmids and transposons through clustered regularly interspaced short palindromic repeats (CRISPR) and their associated proteins (Cas). The CRISPR-Cas system contributes to adaptive immunity, and this acquired genetic immune system of bacteria may contribute to moderating the microbiome of chronic periodontitis. The current research examined the role of the CRISPR-Cas system of red complex bacteria in the dysbiosis of oral bacteriophages in periodontitis. Whole-genome sequences of red complex bacteria were obtained and investigated for CRISPR using the CRISPR identification tool. Repeated spacer sequences were analyzed for homologous sequences in the bacteriophage genome and viromes using BLAST algorithms. The results of the BLAST spacer analysis for *T. denticola* spacers had a 100% score (e value with a bacillus phage), and the results for *T. forsthyia* and *P. gingivalis* had a 56% score with a pectophage and cellulophage (e value: 0.21), respectively. The machine learning model of the identified red complex CRISPR sequences predicts with area an under the curve (AUC) accuracy of 100 percent, indicating phage inhibition. These results infer that red complex bacteria could significantly inhibit viruses and phages with CRISPR immune sequences. Therefore, the role of viruses and bacteriophages in modulating sub-gingival bacterial growth in periodontitis is limited or questionable.

## 1. Introduction

Periodontal diseases are polymicrobial immune–inflammatory diseases that can severely destroy the periodontal ligament and adjacent supportive alveolar bone [1,2]. They are prevalent worldwide, affecting large populations, and have become a public health concern. Dental biofilm is a forerunner in the development of periodontal disease. The sub-gingival microbiota contain more than 700 bacterial species [3,4]. However, the red complex includes *Porphyromonas gingivalis*, *Treponema denticola* and *Tannerella forsythia* (formerly *Bacteroides forsythus*), encompassing the most critical pathogens associated with human adult periodontal diseases [1,5]. Furthermore, the prevalence of potential periodontopathogens, including *Fusobacterium nucleatum*, *Prevotella species*, *Eikenella corrodens*, *Peptostreptococcus micros* and *Campylobacter rectus*, are enhanced in deep periodontal pockets [6], which leads to the spread of microorganisms to the distant site, causing cardiovascular disease, pulmonary infections, cancer initiation and promotion, pre-term low birth weight, Alzheimer’s disease and Parkinson’s disease [7].

Gingivitis and periodontitis destroy the surrounding structure of soft tissue and the hard tissues of teeth, leading to tooth mobility and loss. Diagnoses are based on clinical, radiographical and microbial investigations [8]. These investigations can identify and isolate specific microorganisms or whole metagenome profiles in infected oral cavities. However, isolating and identifying phages and viromes is not a regular clinical investigation. Additionally, the role of viruses and phages in periodontal disease is still debatable, as they have been proven clinically, but their exact role has not been discussed scientifically.

Virulent and temperate phages are two types of bacteriophages. They are viruses that infect bacteria and replicate using bacterial replication mechanisms. A virulent phage, or lytic phage, strictly follows a lytic cycle. In this cycle, the phage infects the bacterium, reproduces new phages using the bacterial machinery and ultimately causes the bacterial cell to lyse (break apart), releasing newly produced phages. This cycle eventually leads to the destruction of the bacterial cell. An example of a virulent phage is the T-even phage [9].

Temperate phages, conversely, can adopt either a lytic cycle similar to virulent phages or a lysogenic cycle. Instead of immediately killing the host cell in the lysogenic cycle, the phage integrates its DNA into the bacterial chromosome and becomes a prophage. The prophage DNA replicates passively along with the bacterial DNA during regular bacterial cell divisions. Under certain circumstances, such as when the bacterium is under stress, the prophage can be induced to switch to the lytic cycle [10].

Temperate phages can play a significant role in bacterial infection and in the ability of a bacterium to escape immunity and cause infection. One of the ways this occurs is through a process known as a lysogenic conversion, in which temperate phages integrate into the bacterial chromosome as prophages and can carry and express virulence factors. These virulence factors can enhance the bacterial host’s ability to cause disease, increasing their pathogenic potential [2].

Another mechanism by which temperate phages can enhance bacterial virulence is through phage induction. Phage induction is a process in which a prophage is excised from the bacterial chromosome and enters the lytic cycle, facilitating cell lysis and the production and release of virulence molecules. This not only leads to the spread of the phage but also aids in the dispersion of virulence factors, posing a potential risk to human health [1].

Emerging technologies such as genome sequencing and transcriptomics have helped researchers dig deeper into the complex interplay between temperate phages and bacterial virulence. They have revealed more subtle ways that prophages can contribute to bacterial pathogenicity, such as influencing bacterial gene expression or metabolic processes. However, research in this field is ongoing, and many intricate layers remain to be explored about the relationships between temperate phages and bacterial virulence.

Recent studies have reported the diversity of viruses in the oral cavity; most viromes contain bacteriophages [11]. Bacteriophage-based therapeutics are currently under investigation for different diseases, as they bypass the problem of antibiotic resistance. In periodontitis, bacteriophage-based therapy can also be applied, as it can overcome the bacteria’s multi-drug resistance and the disease’s recurrence [9]. However, acquired resistance against the phage limits the therapeutic potential of bacteriophages [12]. In most bacteria and archaea CRISPR-Cas (clustered regularly interspaced short palindromic repeats), the system contributes to adaptive immunity in most bacteria and archaea via a DNA-encoded, RNA-mediated and nucleic acid targeting mechanism [13]. Different types of CRISPR-Cas systems have been identified, each with unique characteristics. For instance, the CRISPR-Cas13a system, a type VI-A system, targets messenger RNA rather than DNA. This system effectively inhibits certain phages, protecting the bacterial cell from infection.

Similarly, the type I-C CRISPR-Cas system has shown activity in inhibiting phage antagonists, providing a certain level of immunity to the bacteria. However, this immunity was found to be limited in a study conducted on *E. lenta*, a species of bacteria, indicating that the efficacy of the CRISPR-Cas system can vary depending on specific factors, such as the type of bacteria and phage involved [14].

Although phages are the primary targets of CRISPR-Cas systems, these systems can also target other genetic elements, such as integrative conjugative elements (ICEs) [15]. It was found that more than 80% of isolates with an active CRISPR-Cas system have spacers (segments of foreign DNA stored in the bacterial genome) that target ICEs or similar elements.

Clustered regularly interspaced short palindromic repeats (CRISPR) belong to a family of DNA sequences derived from bacteriophages and are characterized by short, direct repeats separated by spacers. The CRISPR-Cas adaptive immune system, which provides immunological memory by introducing short DNA sequences from phage and other parasite DNA elements into CRISPR loci on the host genome, is present in about half of all bacteria. In contrast to the fast evolution of CRISPR loci in their natural environments, bacterial species normally develop phage resistance through phage receptor mutations or deletions [16]. CRISPR and CRISPR-associated (Cas) genes confer resistance to exogenous sequences of bacteriophages/viruses. Their recognition depends on the similarity between sequences of targeted phage DNA segments and the spacers [17]. CRISPR-Cas systems are present in about 45% of bacterial species and in 80% of archaea. Structurally, the CRISPR-Cas system consists of a group of repeats interspersed by spacers, which are short DNA stretches along with a set of Cas genes in proximity [18]. Immunity is built by gaining short stretches of interfering nucleic acids into CRISPR loci as ‘spacers’ [19]. These immune markers are transcribed and processed into small non-coding interfering CRISPR RNAs (crRNAs) that guide Cas proteins toward target nucleic acids for the specific cleavage of homologous sequences. A new spacer is always added to the AT-rich leader site of CRISPR, which is thought to include unique sequence features for direct spacer DNA insertion [20]. Although the search results provided do not explicitly mention the interaction between CRISPR systems and phages in the context of periodontal bacteria, it can be inferred that the CRISPR system functions as a defense mechanism against phages in these bacteria, similar to its role in other bacterial species. Moreover, developing CRISPR-based therapeutics against periodontal bacteria may also involve strategies that leverage this system’s anti-phage properties.

Machine learning has been used in multiple ways to improve the efficacy and accuracy of the CRISPR-Cas system. One such application is being developed with a deep learning model known as CRISPRon. A study utilized a machine learning model to classify CRISPR arrays. This step is part of a broader CRISPR identification pipeline, which is used to identify potential targets for CRISPR-Cas-mediated gene editing. The Extra Trees classifier from the Python Scikit-learn package was integrated into this pipeline to classify CRISPR candidates.

The current study aimed to investigate red complex bacteria’s acquired phage resistance marker profile via the genome analysis of patient samples. Further, this study attempted to identify spacer sequences, and spacers were BLASTED against the bacteriophage database to identify homologous sequences in phages with machine learning. Additionally, the present study aimed to identify the role of CRISPR-Cas in the phage resistance of periodontal red complex bacteria with a machine learning model.

## 2. Materials and Methods

The study protocol was approved by the institutional ethical committee (IHEC/SDC/FACULTY/PERIO/020), Saveetha Dental College. Five plaque samples of periodontitis patients were sent to a lab for identification. Later, whole-genome sequences were obtained from the NCBI NR sequence database of *P. gingivalis*, *T. denticola* and *Tanerella* [21].

The genomic query sequence, in FASTA format, was the input for the crispr.i2bc.paris-saclay.fr CRISPR tool. Potential locations of CRISPRs, including at least one motif, were identified by finding the maximal direct repeats. The CRISPR pattern of two direct repeats and one spacer was considered a maximal repeat, and repeated sequences were separated by a sequence of about the same length. Whole-genome sequences of red complex bacteria were crosschecked for similarities in the NCBI genomic database. Once matched, the sequences were analyzed for CRISPR using the CRISPR identification tool (University of Paris; CRISPR.i2bc.paris-saclay.fr, accessed on 2 April 2023) [20]. Homologous sequences were obtained and confirmed using the NCBI BLAST algorithm according to standards [21]. CRISPR sequences were identified using CRISPR identification tools [21]. After obtaining the results, spacer sequences were downloaded for each bacterium, analyzed and crosschecked for homologous sequences in phage and virus genomic databases using the BLAST algorithm.

Predicting CRISPR sequences from spacers using AI can improve the efficiency of identifying and characterizing CRISPR systems. The BLAST results were added to each sequence as a separate class if necessary [22,23]. This class prediction was made by using the Orange machine learning tool. Orange supports file loading, transformation and explorative analysis and addresses all the essential phases of necessary frameworks. The pre-processing process includes cleaning the data and preparing the data. In this stage or step, we cleaned and arranged the data that we obtained. We identified a set of missing data in these data, for which the missing features were removed and outliers were removed and normalized, and the data were split into training and test data with 80/20 percent and cross-validation of 20. Machine and deep learning algorithms, such as SVM, Random Forest and Neural Networks, were applied to the CRISPR sequence dataset.

### 2.1. Neural Network

Artificial neural networks (ANNs), modeled according to how biological nervous systems process information, comprise interconnected components called neurons that process and work together to find answers to particular problems. Similar to humans, ANNs base their learning on examples. Instead of a list of guidelines for carrying out a specific task, they are given examples to analyze and devise a solution.

### 2.2. SVM

Support Vector Machines (SVMs) are supervised learning models used in machine learning for classification and regression analysis. They are associated with learning algorithms that analyze data to find patterns and predict outcomes.

SVMs are particularly effective in high-dimensional spaces, where the volume of features (or variables) in the data is high. Even in cases in which the number of dimensions (features) exceeds the volume of samples (individual data points), SVMs can still provide an effective analysis.

### 2.3. Random Forest

Random Forest is a supervised learning method used for classification and regression tasks. It works by generating plenty of decision trees during training. The term “forest” in the name represents an ensemble of decision trees. The main principle behind Random Forest is that a combination of learning models (in this case, decision trees) increases the overall result. Hence, for a more precise and reliable forecast, Random Forest constructs and combines many decision trees [11]. An uncorrelated forest of decision trees is produced using the Random Forest algorithm, an extension of the bagging method that uses feature randomness and bagging. A random subset of features is produced with feature randomness, which adds to the diversity and robustness of the model.

### 2.4. AUC-ROC Curve

The classification model’s performance metric is AUC-ROC. The AUC-ROC metric shows a model’s class-distinguishing ability. As the AUC becomes higher, the model becomes better. AUC-ROC curves graphically show the trade-off between sensitivity and specificity for every possible cut-off for a test or combination of tests.

Classification models are evaluated using AUC-ROC. The AUC-ROC metric can determine a model’s ability to distinguish classes. Models with higher AUCs are better. AUC-ROC curves are widely used to visually depict the relationship between sensitivity and specificity for each conceivable cut-off for a test or collection of tests. One way to assess a model’s accuracy is the area under the curve. A good model has an AUC near 1. A model with a low AUC has the worst separability.

Precision should ideally be 1 (high) for a good classifier. Precision becomes 1 only when the numerator and denominator are equal, i.e., TP = TP + FP. This also means that FP is 0.

Precision = TP ÷ TP + FP

The recall should ideally be 1 (high) for a good classifier. The recall becomes 1 only when the numerator and denominator are equal, i.e., when TP = TP + FN, which means that FN is 0. As FN increases, the value of the denominator becomes more significant than the numerator, and the recall value decreases.

Recall = TP ÷ TP + FN

Therefore, the ideal precision and recall for a competent classifier are 1, implying that FP and FN are 0. Therefore, we need a statistic that considers both recall and precision. The F1-score is a statistic that considers both precision and recall: [(Precision × Recall)/(Precision + Recall)] × 2.

## 3. Results

### 3.1. Identification of CRISPR in P. gingivalis

*P. gingivalis* sequences were analyzed for CRISPR sequences with the identification tool, and the results show *P. gingivalis* TDC60 DNA and the complete-genome CRISPR ranking for the following sequence: 6 Crispr_begin_position: 218514 → Crispr_end_position: 2189300.

### 3.2. Treponema Denticola CRISPR

*Treponema denticola* chromosomes and the complete genome were analyzed for CRISPR sequences with the CRISPR identification tool (CRISPR ranking for the following sequence: 6 Crispr_begin_position: 367189 → Crispr_end_position: 370884).

BLAST—RESULTS Bacillus phage 34.2.

100% query cover; e value: 018, 100%.

The results of the above red complex organisms with the identified CRISPR query cover, spacers and BLAST were determined for sequence similarity and for the identification of microbes.

## 4. Discussion

The immune–inflammatory disease periodontitis can destroy periodontal ligaments and adjacent supportive alveolar bone. Increased oral biofilm buildup, oral inflammation, the recession of gingival tissues and the destruction of the periodontium are symptoms of periodontitis [2]. It is primarily caused by red complex bacterial infections. Red complex bacteria include *Porphyromonas gingivalis*, *Treponema denticola* and *Tannerella forsythia*, which are highly invasive and secrete huge amounts of proteases and proteinases that degrade the host’s collagen and destroy host immune cells, such as neutrophils [24]. These bacteria are located in periodontal pockets and lead to the destruction of periodontal tissues. Several treatment modalities have been employed to treat periodontitis, including antibiotics, pre and probiotics, lasers and ozone therapy, which have shown satisfying results [25,26]. Periodontal disease is also associated with many systemic diseases, including the risk of cardiovascular disease, rheumatoid arthritis and cancer [27,28,29].

Apart from the bacteria, the oral microbiome comprises several archaea, protozoa and viruses and is one of the most dynamic microbial communities in the human body. Dysbiosis of the oral microbiota can affect the host’s immune system and potentially increase periodontitis incidence. Phages are the most common virus in the oral cavity, as is well known. Even though some phages have the virulence to infect and eliminate the periodontitis pathogen, they can survive in the phage-rich environment. Understanding the molecular mechanism underlying phage resistance in periodontopathogens can enable the clinical management of periodontitis more effectively [30]. In the periodontal pocket, bacteriophages are prominent viruses, and the CRISPR-Cas system in the bacterial system might protect the red complex bacteria from these bacteriophages [31]. Clustered regularly interspaced short palindromic repeats (CRISPRs) and their associated proteins (Cas) confer adaptive immunity systems in bacteria and archaea against foreign elements such as bacteriophages/viruses, plasmids and transposons. A CRISPR-Cas genetic structure comprises a series of repeats separated by spacers and a set of Cas genes nearby. In addition to defending against bacteriophages and mobile genetic elements, CRISPR-Cas appears to affect bacterial dormancy, stress, pathogenicity and immune system evasion [32,33]. The CSRISPR-Cas analysis revealed that *P. gingivalis* selectively acquire DNA sequences for their survival and provide protection against foreign RNA and DNA [34]. It has been shown that the virome from the sub-gingival biofilm is distinct from the healthy and periodontal disease state, which implies that the bacterial population might influence phage survival in the oral cavity [35]. We aimed to understand the role of CRISPR-Cas in mediating the phage resistance of red complex bacteria. We hypothesize that CRISPR-Cas systems in red complex bacteria could help protect themselves in the periodontal pocket environment, where bacteriophages are abundant. In addition, the system also helps the bacteria to inhibit the growth of bacteriophages/viruses implicated in the biofilm community.

**Figure 1:**
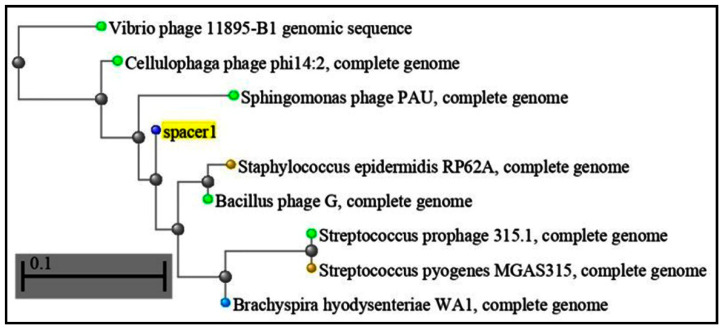
Given homologous cellulophagia query cover of 56.5%; e value: 0.2 s, 100% (BLAST RESULTS).

**Figure 2:**
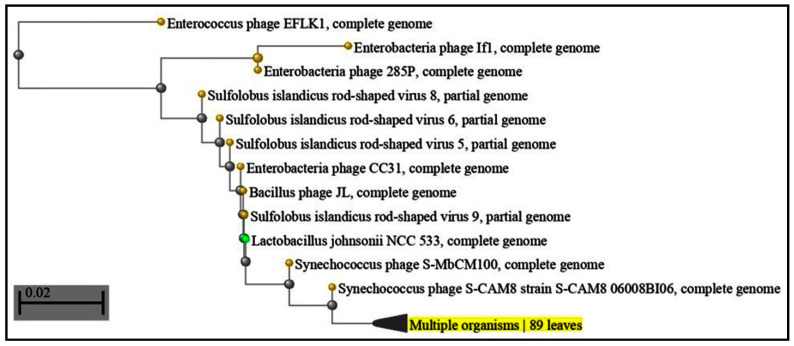
BLAST results.

**Figure 3:**
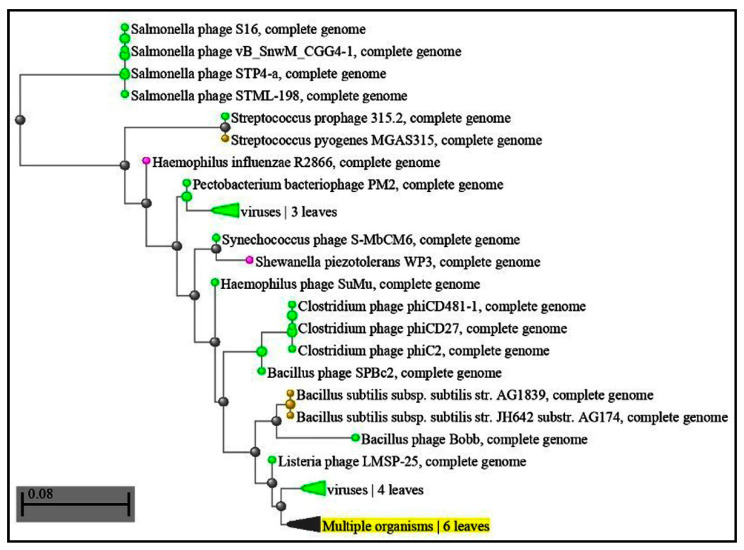
BLAST results similar to those of the pectobacterium phage with an e value of 0.001%; 100% query cover 60%.

Regarding CRISPR-Cas systems in the genomes of red complex bacteria from periodontally affected subjects, our study, which utilized a query sequence of all three bacteria with more than 50 percent, is sufficient to prove repeated attacks of bacteriophages in bacteria. CRISPR gained red complex bacteria sequences that constantly fought phage communities in the sub-gingival microenvironment [31]. More than 2000 oral phages have been reported or are expected to infect species of the phylas Actinobacteria, Bacteroidetes, Firmicutes, Fusobacteria, Proteobacteria and three more (few phages only). The role of phages in periodontal disease has been proven by various studies [36,37], especially cellulobacteria phages Figure 1 and Table 1, bacillus phages Figure 2 and Table 2 and pectobacteria phages Figure 3 and Table 3. Because the CRISPR space is complementary to these phages, the presence of cellulophages (e value: 0.2), (*P. gingivalis*), bacillus phages (e value: 0.18) (*T. denticola*) and pectophages (e value: 0.001) (*Tannerella*) indicate that these bacteria are in a constant fight with phage communities [19]. Zhou et al. found larger Shannon–Wiener diversities of DRs in periodontal disease than those in healthy periodontia, but not for spacer composition [32]. This may imply that healthy persons have a robust bacterial population resistant to phage invasion. The concept of viral modification in bacterial and host environments in the periodontal sub-gingival microenvironment must be revisited, as these bacteria have developed adaptive immune CRISPR spacer responses [38,39].

**Figure 4:**
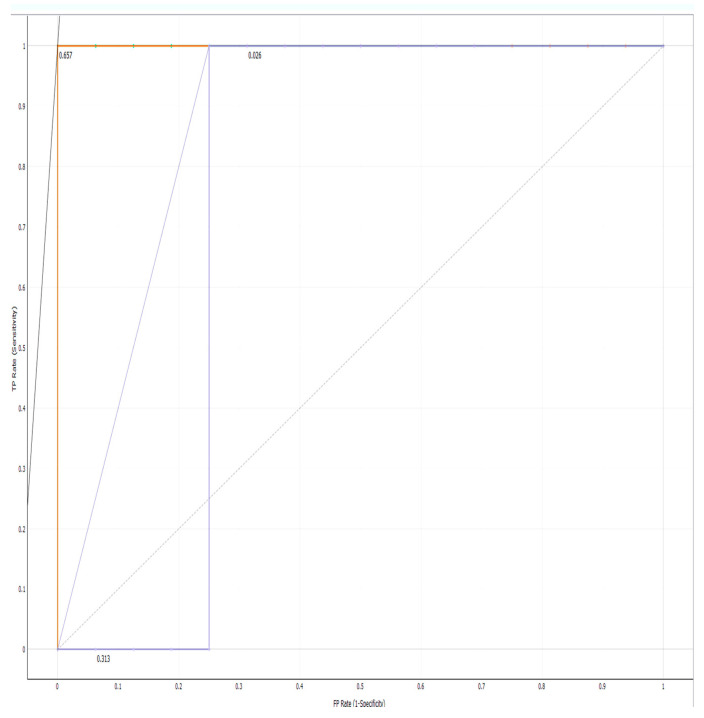
ROC curve of CRISPR-predicted sequences.

Machine learning is a rapidly developing technology today. It is used for image recognition, speech recognition, traffic predictions and product recommendation, and now, this technology is developing in medicine. CRISPR-Cas systems identify and destroy intruder DNA by matching spacers to viral protospacers [40]. These CRISPR array spacers can also be predicted. Modeling CRISPR sequences reveals accurate predictions of 100 percent for Random Forest, Neural Networks and Support Vector Machines. (Table 4, Figure 4) This prediction can help identify and classify red complex spacers in future studies.

The application of phage therapy in dentistry is still in its infancy and requires exploration. Bacteriophages are against many bacteria that are present in biofilms [41]. Understanding bacteria–bacteriophage interactions and specificity is the first step in expanding its applications in dentistry. In this study, we aimed to identify the bacteriophages interacting with red complex bacteria in periodontitis. The formulation of phage-based cocktails derived from different phages could surpass bacterial resistance against a single bacteriophage. Phage-based products can be developed based on the virulence of the phage against the bacteria and the profile of the CRISPR-Cas of the bacteria [42,43]. In that way, our study is the first step toward understanding the therapeutic possibilities to treat periodontitis. Our results show that the CRISPR-Cas of red complex bacteria target phages such as cellulophages (*P. gingivalis*), bacillus phages (*T. denticola*), and pectophages (*Tannerella*) in periodontitis. Our results suggest that red complex bacteria are resistant to phages such as cellulophages, bacillus phages, and pectophages in periodontitis. There are currently only a few clinical uses for CRISPR in periodontics. However, the possible clinical use of CRISPR is to target the periodontal biofilm and create new methods for lowering or getting rid of periodontal infections. Additionally, CRISPR can change the transcriptome and gene expression of genes that contribute to the development of periodontitis.

## 5. Conclusions

The current study highlights that viral phage communities cannot modify sub-gingival bacterial environments, as they have acquired immune mechanisms via CRISPR-Cas to kill the virus and competitively infect periodontal pockets. Future research can be simplified, reducing time and effort by using predictive modeling of red-complex-based spacer analyses.

## Figures and Tables

**Table 1:** CRISPR arrays with spacers of *immunological memories that resist future infections using spacers, or viral DNA fragments for P. gingivalis*. Homologous sequence. Identification using the BLAST algorithm with the bacteriophage database.

Spacer_Begin_Position	Spacer_Length	Spacer_Sequence
2181514	36	AAACGAAATGAAAAAGACAACAAACAGAAGACCCTC
2181580	36	GTGCCAGCTGCAGGGGGATGACATAGCCATTGACGA
2181646	36	GTGCCAGCTGCAGGGGGATGACATAGCCATTGACGA
2181712	36	TCCGCGCCGCGAGGTGGAGACCCTGCCGGAGGCGAA
2181778	37	CCTGAGAAAGAGGGGAGGGAGGAGCGATAGACGAAGT
2181845	36	CGAGAATCTATTGAGTAGCGAAGTCGTCACAAAGAT
2181911	36	AGCCATAGCTCTTCAATTTCAATTTCTTCTTTTAAT
2181977	36	CGAAAATAACAAAAATAGATATATTTATAAAAAAGA
2182043	34	ACTCTTATCATCTACTATCTCAAAAGCTCTTTTT
2182107	38	TCGCTATAACCCTATGTGATTCAGGAATCGGCTTGCTA
2182175	36	GAAACATTCGAGCCGTATTCAATTACGCCATCAATG
2182241	38	CACCCATTGTGCCGCCGTCCTGACCGAAAACTTCTTTA
2182309	38	TCGCTATAACCCTATGTGATTCAGGAATCGGCTTGCTA
2182377	36	GAAACATTCGAGCCGTATTCAATTACGCCATCAATG
2182443	38	CACCCATTGTGCCGCCGTCCTGACCGAAAACTTCTTTA
2182511	38	TCGCTATAACCCTATGTGATTCAGGAATCGGCTTGCTA
2182579	36	GAAACATTCGAGCCGTATTCAATTACGCCATCAATG
2182645	38	CACCCATTGTGCCGCCGTCCTGACCGAAAACTTCTTTA
2182713	36	ATTTCTTCATCTCGCGCTTGCTCAAAAGCGCGTTCA
2182779	34	TGCATCAAGTCACGAACTTTCTGCGAGATGGAAA
2182843	36	CCGTCTCGAAAAAATATCGGGACGTTTTTGTTTTCT
2182909	35	CTCCTTCACTTTGTCGACAATGTGCACTGTATTTG
2182974	36	AGGCGGAGTATCTCTTTGCCACCCAGTCCGCGCGCA
2183040	36	CGTAACCGCCTCGGTAGACCGCTCCGCACGGTCGTT
2183106	37	GTGCATTCCGGACAGCTTTCGCTTAAAAAGTTAGCGG
2183173	37	AAGAACGAACGCCTGCGCGATAAGCACCGCGAGCGTA
2183240	36	ATTTACCTGCAGACTTGTGCCCACCCACTTGATAGA
2183306	36	TGGTCACGGAGCGATACCATGAGTGTTTAGTAGATG
2183372	36	CGGAGGAGATCAGCTATGCGGATGATACCACCCGTG
2183438	36	TCAGAAAACTCGCGTCCATCTGATAGATGTACACGA
2183504	36	TAAACGATCGAGGCGCGGAGACCCTCCTTGCCAGTA
2183570	37	CCGTTCAGGAAAAAGTAACCGAGCTGAAGACCATGCT
2183637	35	AGGTGCTATCGCAGGACTGCAGGACATCCTCAATC
2183702	36	AGCCGCTCGACTTGACGCCATGCAAAAACAGATAGA
2183768	36	TCTGCGAGTTGTGAGAGGCAATAAACTGCTGGGAGC
2183834	35	ATTGCTGTTAATTCTGTCATCTCTTATTTCTTTTA
2183899	35	TCTATCTGCGTATAGCCCTCGCCGTCCACGCCCTG
2183964	37	GGGAAGCTGCTTTGCTCGCTGAGATAGCAGCACTTCA
2184031	36	TTTATTGACGCCACCCCGCCGACGAAAAAAAATCAT
2184097	36	TTTCGGTCTTTACGTTTGTCGCCACGGATACATGCT
2184163	37	ATTGTAAATAAATTACATGGCTATTGAAAAACAAATT
2184230	36	GATTGTACGACTTTGCTATAAAGTCTGAGTTATTAA
2184296	36	TATCAACAATCACCGTCATATGTGTAATATACTTGA
2184362	36	GGGCAGGCGTATTGCCCCACTTCTCCCCGAATGCAT
2184428	36	TGAGGAATCATATCAGTGTTTATTTTTTCATCGATT
2184494	36	TGGAAGTGGGTAGAAGAAAGCCCCAACGTGTTTAAG
2184560	36	CATGACAAAGAGACGGTTATCGGTAGAGGACAGGCA
2184626	35	CGCGTGGAAGGGGCATGTACACTTGTAGTTCGCCC
2184691	34	TGACAGGCCCTGCAGCGTGTGAGAGCGGAAATGC
2184755	37	ATTTTCAATCATGATATTTTATTTTTCCTGCAAACGC
2184822	36	TCGCATGTGGGAGCGCGGCGGTCTCTGCTTTACGAA
2184888	36	TCAGCGTGATGAGCGCTTGAGGCTCCTCCATCGAGT
2184954	36	AAGGATGATTTGGAAAATTTAGTAAGATAGTTGATA
2185020	37	TTCTTGGAGAAAGCGAAGACCATGAACCTAAGCGTCG
2185087	36	GGAAATATAGTTATTGTATCTACTAAAAGACATAGT
2185153	34	ACCCCATCTTGCAGAGTATATGCGAGCAAAATTT
2185217	34	TAGTTAGCACAGTTGCTACTATCGTAGTAGCTGT
2185281	35	AGATAAACTTTCTTCTCGAATTAAGAAAATCGAGA
2185346	36	CCGCGGCCATCGAGGCCACCGCCTCCGTCCTCCGCG
2185412	37	TATGAAAACAGAAAAGAACTTCTCAGCCCTGAGTTTG
2185479	37	CAAACTCAATGATTATCTGTCAAGAAGCAAGAAACGA
2185546	37	AGTAAAAATTACCCTAGATGCCGAAACGGACGGCCTT
2185613	36	CAGAATTTGCACGAACAGTATGATGTTCGTGTTCTT
2185679	36	AAGCGCGAGACAGGCCGAGCCGGCACAGCTTAGTGC
2185745	36	AAAACGGCGATAAAATAGCGTTCGAGATTTTCCGCA
2185811	37	TAGTTGTAGCGATTGTCTCAGTTGCATTACTCCTTAC
2185878	37	AAATAACGAGAAAAAGAATGCTTAAATTGTTCTTCGT
2185945	36	AGAAGAGGGTAAACTATTTGCTAATCTTGAATGCTT
2186011	36	TCTCAATATCTTTCATAGCTACTAAAAATTTACGAA
2186077	35	ATTGCTGTTAATTCTGTCATCTCTTATTTCTTTTA
2186142	35	TCTATCTGCGTATAGCCCTCGCCGTCCACGCCCTG
2186207	36	TGCCCCCTCACCCATCTAACCTCGAGCCGTTGAGCC
2186273	36	GATTACATAATGATAGACGACAGAGATTGTGCAGAA
2186339	35	GTACTGATAATTACGCTGCAAGGTCAGACGGTGAT
2186404	36	TTGCCAGGGCTTGCTGATGCGCGCGCTCCAGCTGCT
2186470	36	ACAGAACCAGCTCCGTCAAATCTCCCGCTTTTTGTC
2186536	36	TGCCCCCTCACCCATCTAATCTCGAGCCGCTGAGCC
2186602	36	TATTCATTCGCTCAAGCGAGGGCATCCTGCTGCAGC
2186668	36	GCTCGCGGAGGGAGAGGGTGCCGGTATCCTGCCTCC
2186734	36	AGAGTCTGTATGTGAAAGTGTAGTTCGAAACATTAT
2186800	34	AGAATACTTTGAAGTTGTATTCAATTACTTTGAA
2186864	36	CCGTTGTGACAGAGCTGCGCCGTCGTGGCTATGATG
2186930	36	TGAAAGCCCGAAGATAATCTACACGCAAGATTGTTA
2186996	35	CGTCATCAGGTGGATATTCTTACTGCTATCCACGA
2187061	36	TCGTCTGCGACGTATCGCAGCTCTGCCAGCTCCGCG
2187127	36	GCATCTTCGCAGGAAAGAAGAAGGCTCCGTCCTCGA
2187193	36	AACAGTCAAGGGGGAGCCGACCTCTCCGGCGGATTT
2187259	36	GCAGTGCGGCCGACAAGGCTAACCTCGCTCAGCTGA
2187325	36	AATCCATGTTCATGAGGAGAGGATACGAGTTCTATT
2187391	35	CCTTTTGCTATTGCAGCAAACATATACAATAATAA
2187456	36	ACTTCATGGATTTAGCGAGATACTCATTATAATTAA
2187522	36	TCTTCAAAGATAGTTGTTATCAAATATCGCGCTGAA
2187588	37	ATCTTCAAAGATAGTTGTTATCAAATATCGCGCTGAA
2187655	36	GCTCAAGCGTCCAAGAAAGTTCAAGAAATTGTACTA
2187721	37	TACTATGGTGTCTGCATTTGAGATACGCAAATAGCAA
2187788	36	CCTCAATATCTTTCATTGCGACGAGAAATTTGCGAA
2187854	36	AATCTATGATGATATAGAAGAAGACGACTTGTTGTT
2187920	36	ATTAATGATTTTCTGAAATAAAGAAGCAGTTGCATA
2187986	35	CTCAATATCTTTCATTGCGACGAGGAACTTGCGAA
2188051	36	CCCCGTGGTAACTCATACCACCGACTATTCCACCGT
2188117	36	TTTGAGTAATCATCGAATAATTATCGATTAATCATT
2188183	36	TTATATGCATCATATTCTTAAAGTATTTTATTTGAA
2188249	36	GACGAATACGGCGTTCATCATCGATAGTCGCGATGC
2188315	36	CGGGCGGCCAACCGGTCACAACAAGAATAGACCGAT
2188381	35	TTCCTTCCAGTCGCAGCTTAAGATACTGCGACTAC
2188446	36	AATTTTCATCAGAGCATAAAAAAGGGCAAACTTTTT
2188512	35	GATACGAGCACCAAGGCTGCGATACCGATTGCGTA
2188577	36	CCTCGAGCAGATCTTCCTGCTCTTTGATGAGTGAGG
2188643	35	GCAGGTTGATTATAATGTTGAAGATGCTTTAAGGG
2188708	36	CTCGTATCGACTTTCAAGCAGGCTGGAGTGCAGCCT
2188774	37	AATATGAGATCGGAAACAATTATAGTTGCGTCGATAT
2188841	37	TCACAATAACCGACAAAATGTCTCGCGTAACGTACAA
2188908	35	GTCGCCTGTTTTCTTGAACTCCTCACTGATTCGTA
2188973	35	GATGGTGTCGACATCATACGACAATAGATCGTCGA
2189038	35	GATGGTGTCGACATCATACGACAATAGATCGTCGA
2189103	36	AATATTTTTTCAAAAATTGTAAAACTTATTAAGTCA
2189169	36	AAGTTGCTGATTGTCTTAGAATGAAAGGTTATGCTC
2189235	35	ATAATATTACATAATGGCACACTGATGGTAAACTT
2189300	34	CATGATGTACAAATATATCATGATCGTATACTAC

**Table 2:** The CRISPR array identified with sequences of *T. denticola*.

Spacer_Begin_Position	Spacer_Length	Spacer_Sequence
367189	30	TATAGGAGGTTTCAAAATGGAAAAATCGAA
367255	30	TATCAAGTTGAGCCTTCTTTAAAGCTCCGC
367321	30	TATAGGAGTTCCAGACCCAGCACCATCACC
367387	30	AAAATCGAATGTATCGCAAGATTCAAACCA
367453	30	TACAAAATCGAAGCAGAAGAAAGGAACTTC
367519	30	GGTTCCAATCTTTTTGGAATGATTAACAAT
367585	30	GATTCTGTATTTCAACGCGATGTTGCTAAT
367651	30	CTAACAAAAGGTGGAATTTTACCGAACAAT
367717	29	AATTAGTTGTCATTGAAGGTGAAGCCGGA
367782	30	GCGGAAAAACTATATCGTAATCTTCATAGA
367848	30	GCTGGAACGCCTATAGCGACGCAAGCTCCT
367914	30	CGCTGGAACGCCTATAGCGACGCAAGCTCC
367980	30	GGTTCCAATCTTTTTGGAATGATTAACAAT
368046	30	CATCTAGAATCCTATAAGGCACGAAGTAAT
368112	30	CCTTTTTTGTAACTCCTATTTGCAGCTATG
368178	30	ATTACTTTTCGAAAAAAAGCCGTATTATAG
368244	30	TCTTTGTATTATAAAGTTAGCAGAGGAAAA
368310	30	GAATCTACCACCCTCAATACTCCGCCTATT
368376	30	GTCAACATCACCGCGATCACTACAAACAGC
368442	30	GAATGAAAAGGACAAGGAAAAAGCTGCCCT
368508	30	TGATTATTTGGAAGGCATGAGTAAATGCTG
368574	30	GCAGTAACTCACAAGCCACTTTGAGAGTTG
368640	30	TTCGACGCTTGTCGAAAAGGCAATCAAGGC
368706	30	CGAGAAGTTATTATTCTGAACTTCACATCG
368772	30	CTTTGGTATCAATTAGGATTTCCTAAAGTC
368838	30	TACAATGATTGCTTGTTGTTCTGATGGAAC
368904	30	TAGCCTCACCATTATAAAGCAATTCGCATG
368970	30	TGTTACGTCAAAAAATCCAATAAGTTGAAG
369036	30	CCTGATAAGGAAGATTGGCGAAAGAAGGTA
369102	30	TGCTACATCAAATAACCCTACAAGTTGAAG
369168	30	CCAAAAGTTCACAGTCATCCGAGTAGACGT
369234	30	CTATCTACTTTTGGGAACCCTAATTGGTAC
369300	30	TTTCTTCTGTTTGTCCATGTCCAAACCTCC
369366	30	AACAATGTGTGATTTTTCGGACTTAGTCCC
369432	30	AAGGGAATAACCTTACCATTCTGTCTTATG
369498	30	TTCCCAAAAGTTGATGCTGATACGATTGGT
369564	30	AACAATCAGCCGTGAGGGAATACGCCGCGT
369630	30	AGGTTAATGATGAAAAAAATAATAACTACT
369696	30	GGGCATATTATGCAGATATGCAACGAAACG
369762	30	CTTGGAAAAGAATTTATAAAATGCGAAGTT
369828	30	GAACATATGCTCGCTCTTTCTCGAGTACTC
369894	30	AAACTTTGAGGTACTAAATAAAACAAGTCA
369960	30	ACCTTTCAATAGTAGCATCGGGCAAACCAG
370026	30	GTCTCTAGTTACTTTACGTATAAACTCTAT
370092	30	GGGCATATTATGCAGATATGCAACGAAACG
370158	30	CTTGGAAAAGAATTTATAAAATGCGAAGTT
370224	30	ATGCGATATATCTATGACTTTACCTATTCT
370290	30	AAACTTTGAGGTACTAAATAAAACAAGTCA
370356	30	ATATCTTTTGTCGTTAAAGTTAGTAAAAAA
370422	30	TTTGAAATTCCCCAAATGTCAATTGTTTTC
370488	30	GAAAATGCAGGCGGTTCCACTGGAGAGGTT
370554	30	TAATTCAAAAAAAGGTCTTGGTTTGAAAGG
370620	30	AGCCCGCCCTGCGGAATTGCACGGCCCGTT
370686	30	ATTGAGCGTCAAGCACCCGGTAAGCCCACC
370752	30	TTGGTTATTCGACTTTTGATTTGAGCTATC
370818	30	CTCGCTCGAGCACAACAGGTGGCTGTCCAC
370884	30	TTTCCAGCTAGAGCATCAAAGTTTATAGGG

**Table 3:** Identified CRISPR arrays with spacers of *Tanerella forsythia*.

SPACER ID	POSITION	SEQUENCE
2508368	36	ACAGAAACTTCTTTTCCTGCAAGATTAAATAATACA
2508434	37	GGTATGTATAAATCTACACGTCTTGGGTTTTCTAATA
2508501	40	AAAATTATCTTTGATAACTTTAAGAATCTTTTTGTCTTCT
2508571	40	CCATTACTGCGCGGGCGGCGATGCAGGAGAACCCGGACCG
2508641	39	TAAAAGGTTAAAAGTTAAATGAAGAAAGACATAAAACGA
2508710	36	GTCTTTGGAGGCCTTTACTCTTTTAAAAATGCCCGA
2508776	34	TCTCTTGTGTAGTTATAACACACAATTGAGTCAT
2508840	35	CCTCTTCGTAATACGGCTCTATATCGAGCTCTCTG
2508905	38	CCCCGAAGGCGCGGCCGTTCCACTCTAAGGTGTCGACT
2508973	36	GTTAGATGATAACTTCCGTCTTCTCCGAGCATCATC
2509039	36	TTCACGGGGGTAAAGCCCGCCCCTTACGGGGAACTA
2509105	37	AAAAGATTTGCTATATTGTGAAAAATTTAAAAAAAAG
2509172	39	TTCACGGGGGTAAAGCCCGCCCCTTACGGGGAACTACTC
2509241	38	TGCAGCCGTTGTCTCGCAAAAAGCAGCGCCTTCTAAAA
2509309	36	CCTGCCCTGCGTGGGGGCTTTGGTGTGCGCAGCCTG
2509375	37	CTTCAAAAGCGGCAGCGCGCTTTTTGAGAAGGCGCTG
2509442	37	TTTGTGTTATACGGAGATTATACACGGTGGGTGTGGG
2509509	37	TGCGCGCATACGTTCTTCTACGTACGCCTTTTTGGTT
2509576	38	AAAAGATTTGCTATTTTGTAATAGCAAAGAACTATGAA
2509644	35	TAAGAGGTTATCTCCGTCCGCACGAGTTCGGACGT
2509709	36	CATACGTTGCACGAATGTCCGCATCAAAAGCGGCAG
2509775	36	GTTATTCTCCCAATCACCGAACCTGTCAACAAAAGA
2509841	38	CTATAACTGCAGCCCGCACGGCTGCACGGTTGGCGTAC
2509909	37	GTCTGGTCGTTTTTTCTGTATTGGGGGGGGCGGCATT
2509976	36	ACGTTAACACGTGCACCGCCAACGCGGGCAGAAAAG
2510042	35	CGTATCTGAAACGTCCGAATTGGTGCGGACGGAGG
2510107	36	GCCACCACCGACCCTGCACGGGCGGTTTGGCTCGCG
2510173	37	ACATAGCAAACCTTGTTGGATAATATTCTTCGTCTAT
2510240	36	GAACAATAACAAACAGGATTGGGCGATCATACTTTT
2510306	36	CTCGGATTTATCCATTACGTCGGCCGCATCGATTTG
2510372	38	CCAACGCGGGGAAGAAGGCGGATGAGGTTTCGGAACAA
2510440	37	CGCCGAGAAGGTCTCCGTCCTCGTCAAAAACAGACTC
2510507	37	GCTCGGGGATCGATATAATAGAATGGTAGAGGAGTGG
2510574	38	TACGGGGTCACCTCCGTTCGCATGAGTTTGGACGTTTC
2510642	36	CATATAACAGTGCGACGAGACAGGCTGCGCACACCA
2510708	37	GGGGTAAATCATGTAAAACGAACAATTTTAGAATATA
2510775	36	CGTAGCGCTATTATGCGCGGTATGGCGCGGCTCTGC
2510841	36	CTTGCAGTAGGGGCAGTGAAAATTACACGACCCGAA
2510907	39	CTATTACCGCCGCCCGCACGGGCGCACGGCTGGCGTACG
2510976	38	GAATAAAAATCAGCGTAAAAACATATATGCTTACACGA
2511044	38	TTTTTTGTGGCCGTTTCGGCCTTATCTGTATCTACTGT
2511112	36	AGCCGACGCGATATATTCGTGCCCGGCAAACAAATC
2511178	36	CGCCGTACACGTGGTGATATTTATGAAACAAATTTT
2511244	36	GATATATTTTTCTAAACATTTTCATGACTTTATCGG
2511310	36	TGATAACTTTTAAAGTCTTTTTATCCTCTGCATTCA
2511376	37	ATTGTACACCTTGTCTACTTGCTCATCAGGGAAATTC
2511443	37	GTAATCCTCACACTGCCGATTATGGCTGAGTAATCTA
2511510	36	AGTTGATGCAAAAGGTTTATCGAAACACCTTCCTTC
2511576	37	CCTTATCTCAATGGTTTAAGGAAGACGTTGCGCGCAT
2511643	36	AGCCTATCTAAACGCCAAATAACTAATTTGTCTCCT
2511709	37	TCGGTTTGGTGATAGTTGGTAACAAGATTTTAAAACA
2511776	34	ATTACATCAGGATAGATGGTGTGGCGTATGAAAA
2511840	37	GAAGCTGGTAACCGCGGATGTCGCCTTCGGCCTCGAA
2511907	38	CCGCTACCCAAAAAACCCTCGACAAAATGCCCGAAATC
2511975	37	CACTGGGGCACATAGTCCTGATCTCCTCGGCGAGTAT
2512042	37	GAAAAGTGCTAATGGTAAAGTTAAACTTTCTCCTTTA
2512109	35	TTAGATGATCTTGCGGACTACAGTGCCGAATTTGA
2512174	36	GCAAACGGGGCCTTTTGTGCCATGTCACCAGTGCGG
2512240	37	AGAGACGTTCCTCCTGCGTTGTACAAGATACTCTGTA
2512307	34	AATTTAAGTGATGATGTAAAAACTGGTATTAATA
2512371	35	TTAGATGATCTTGCGGACTACAGTGCCGAATTTGA
2512436	36	TTCGCAATCGCTTCGACGGCAAAAGTGCCGCCCAGG
2512502	35	TGTTTATGTACGACTCTTTCAGTTTTAACTGCTTT
2512567	39	GATATAATGCTTACAATCGAGGCATTTAATAACATTAAA
2512636	39	CAATGTGTACGTTGTCATTGCCGGTATATTAGGATTCTT
2512705	35	AAGTGCCTTTGCCGTTAATTTTGTCAATGAGTTTC
2512770	38	GCGAAATCCTGCTCGTGCAGGGTGGAAGCAAGAATATC
2512838	38	AGCGTCATAGCATTCACACCGGCAGCACCAGTTACGAA
2512906	37	GTCGGACACGATGGGCAGAAATTCTTCTTCGACCATG
2512973	35	CCGTTAAATTGTCTGGCAAGGACGTGACGCCGGTA
2513038	36	CGTTGTAGAAATCGTCTTCGTTGATAACAAGTGTTA
2513104	37	GCTTCTTCGATTCTTCTTTTACCTCTCCGGTTTCCGT

*Tannerella forsythia*: complete genome length, 3405521; CRISPR rank in the sequence, 4; Crispr_begin_position: 2508368 → Crispr_end_position: 2513104.

**Table 4:** Accuracy of nearly 100% in all algorithms.

Model	AUC	CA	F1	Precision	Recall	LogLoss	Specificity
Random Forest	1.000	1.000	1.000	1.000	1.000	0.097	1.000
Neural Network	1.000	1.000	1.000	1.000	1.000	0.021	1.000
SVM	0.938	1.000	1.000	1.000	1.000	0.387	1.000

## Data Availability

Not applicable.
